# Immune checkpoint inhibitor-associated new-onset primary adrenal insufficiency: a retrospective analysis using the FAERS

**DOI:** 10.1007/s40618-022-01845-z

**Published:** 2022-07-23

**Authors:** D. Lu, J. Yao, G. Yuan, Y. Gao, J. Zhang, X. Guo

**Affiliations:** Department of Endocrinology, Peking University First Hospital, Beijing, 100034 People’s Republic of China

**Keywords:** Primary adrenal insufficiency, Immune checkpoint inhibitors, Immune-related adverse event, Immunotherapy, Prognosis

## Abstract

**Background:**

Our study aimed to investigate the prevalence and demographic characteristics of immune checkpoint inhibitor-associated primary adrenal insufficiency (ICI-PAI) and to explore the risk factors of its clinical outcome using data from the US FDA Adverse Event Reporting System (FAERS).

**Methods:**

This was a retrospective study. All cases of new-onset or newly diagnosed primary adrenal insufficiency associated with FDA-approved ICIs from 1 January 2007 to 31 December 2020 were identified and collected using FAERS. Data on age, sex category, body weight of the participating individuals, the reporting year and the prognosis of cases, and other accompanying endocrinopathies related to ICIs, were analysed.

**Results:**

The incidence of ICI-PAI was 1.03% (1180/114121). Of the 1180 cases of PAI, 46 were “confirmed PAI”, and 1134 were “suspected PAI”. Combination therapy with anti-CTLA-4 and anti-PD-1 was related to a higher risk of PAI compared with the anti-PD-1-only group (χ^2^ = 92.88, *p* < 0.001). Male and elderly individuals showed a higher risk of ICI-PAI (male vs. female, 1.17% vs. 0.94%, *χ*^2^ = 12.55, *p* < 0.001; age < 65 vs. ≥ 65, 1.20 vs. 1.41%, *χ*^2^ = 6.89, *p* = 0.009). The co-occurrence rate of endocrinopathies other than PAI was 24.3%, which showed a higher trend in patients on nivolumab-ipilimumab treatment than in those on PD-1 inhibitors (*χ*^2^ = 3.227, *p* = 0.072). Body weight was negatively associated with the risk of death in the study population [*p* = 0.033 for the regression model; B = – 0.017, OR 0.984, 95% CI (0.969–0.998), *p* = 0.029].

**Conclusion:**

ICI-associated PAI is a rare but important irAE. Male and elderly patients have a higher risk of ICI-PAI. Awareness among clinicians is critical when patients with a lower body weight develop PAI, which indicates a higher risk of a poor clinical outcome.

## Background

Immune checkpoint inhibitors (ICIs), including programmed cell death protein-1 (PD-1) inhibitors, programmed death-ligand 1 (PD-L1) inhibitors and cytotoxic T-cell-associated protein-4 (CTLA-4) inhibitors, have demonstrated potent and prolonged antitumor activity in patients with advanced malignancy during the last decade. Immune-related adverse events (irAEs) of ICIs, including endocrinopathies, have been recognized in a subgroup of patients receiving ICI treatment [1]. Primary adrenal insufficiency (PAI) is a type of ICI-related endocrinopathy, second in line with the prevalence of thyroid dysfunction and hypophysitis while preceding type 1 diabetes mellitus (T1DM) [2].

ICI-associated PAI (ICI-PAI) was first reported to the US FDA Adverse Event Reporting System (FAERS) on 21 December 2007 in a 56-year-old female patient receiving ipilimumab monotherapy. In clinical practice, patients with advanced-stage cancer could develop adrenal insufficiency due to ICI-related PAI, ICI-related hypophysitis, or bilateral adrenal metastasis [3]. ICI-PAI could be distinguished from secondary adrenal insufficiency caused by ICI-related hypophysitis when the complete destruction of the adrenal glands, including the zona glomerulosa producing mineralocorticoid, was proven. In addition to elevated ACTH as one of the diagnostic criteria of PAI, the elevation of the renin level and hyperkalaemia induced by adrenal insufficiency could help support the diagnosis of PAI. As a potentially life-threatening subtype of endocrine-related irAEs that can lead to adrenal crisis and death, it is critical for both endocrinologists and oncologists to obtain accurate data from a large population about the incidence and clinical manifestations of ICI-PAI. However, the incidence of ICI-PAI has only been described in a few case reports and it varies among studies [5–7]. We carried out a retrospective study to investigate the demographic characteristics of ICI-PAI and to explore risk factors for its clinical outcome using data from the FAERS.

## Methods

Our study is a retrospective study based on the FAERS database, mainly focused on ICI-related PAI. The FAERS is a public-open database for adverse events induced by all FDA-approved medications. All cases of new-onset or newly diagnosed primary adrenal insufficiency associated with FDA-approved ICIs from 1 January 2007 to 31 December 2020 were identified and accumulated in our study population using FAERS. The ICI medications included in this study were anti-CTLA-4 mono-antibody (ipilimumab), anti-PD-1 mono-antibodies (nivolumab, pembrolizumab and cemiplimab) and anti-PD-L1 mono-antibodies (atezolizumab, avelumab and durvalumab).

New-onset PAI or adrenalitis in patients receiving ICI treatment was considered a diagnosis of ICI-PAI. The definition of ICI-PAI included the following FAERS terms as adverse reactions: “adrenal insufficiency”, “adrenalitis”, and “primary adrenal insufficiency”. PAI commonly focused on the adrenal function when patients presented with elevated ACTH and decreased cortisol, while adrenalitis was diagnosed when patients experienced symptoms of adrenal insufficiency and the image of their adrenal glands was altered. Those with “secondary adrenal insufficiency”, “pituitary adrenal insufficiency” or “hypophysitis” after ICI treatment were excluded from the study population. When there was uncertainty in the origin of the adrenal insufficiency, that is to say, cases diagnosed with “primary adrenal insufficiency” and “hypophysitis” or “secondary/pituitary adrenal insufficiency” at the same time, they were excluded from our study. Since the FAERS is a descriptive database of adverse events, the diagnosis of PAI and the exclusion of secondary adrenal insufficiency were based on the reported reaction terms. Therefore, we defined “confirmed PAI” when the discrimination included primary adrenal insufficiency, Addison’s disease, adrenal insufficiency + hyperkalaemia, adrenal insufficiency + elevated corticotropin, adrenal insufficiency + increased corticotropin, and adrenal insufficiency + elevated renin. The reported ICI-PAI cases that failed to meet the above discriminations were assigned to the category of “suspected PAI”.

Data on age, sex categories, the reporting year of cases, the body weight of participating individuals, other accompanying endocrinopathies related to ICIs and the prognosis were collected. The co-occurrence of a second immune-related endocrinopathy in addition to PAI was defined as a situation with other accompanying endocrinopathies. In the FAERS, patients listed with the outcomes of hospitalization, disabled, life-threatening and death were considered severe cases; otherwise, they were assigned to nonserious cases. These labels, including hospitalization, disabled, life-threatening and death in FAERS, were due to all reaction terms that the cases were labelled with, possibly involving causes other than “PAI”. Since all cases in the study were anonymous and all data are available in public resources, this study was exempt from informed consent and ethical approval.

## Statistics

Quantitative variables with a normal distribution were analysed using one-way ANOVA. The proportion of ICI-PAI among the total ICI-related adverse events during the same study period or in the same population was a quantitative variable and was compared using the *χ*^2^ test by sex category, age and reporting year after applying the Bonferroni adjustment method. We used logistic regression analysis to evaluate the risk of a poor prognosis after adjusting for confounders, including sex, age, body weight and subtypes of polyendocrinopathies, among patients receiving different regimens. Statistical analysis was performed using SPSS 17.0 (IBM, U.S.). *P* < 0.05 was considered statistically significant.

## Results

### Incidences of ICI-PAI in all cases with adverse events

In total, 1180 cases of ICI-PAI were identified in 114,121 patients reported as adverse events related to ICIs, and the incidence of ICI-PAI was 1.03%. Of the 1180 cases of ICI-PAI, 65 were categorized as “confirmed PAI”, and 1115 were categorized as “suspected PAI”. The distribution of sex categories, age, body weight, ICI regimens, cancer types, accompanying endocrinopathies and clinical outcomes between patients with confirmed PAI and suspected PAI were not significantly different (Table 1). Due to the comparable results, we conducted further statistical analysis of all reported 1180 cases of ICI-PAI.

**Table 1:** Clinical characteristics of patients with “confirmed PAI” and “suspected PAI”

Characteristics	Confirmed PAI (*n* = 65)	Suspected PAI (*n* = 1115)	*p*-value
Male/Female/Not specified	35/25/5	703/334/78	0.319
Age (year)	61.9 ± 12.4	65.1 ± 10.8	0.065
Weight (kg)	74.0 ± 22.5	72.3 ± 21.4	0.674
PD-1/PD-L1/CTLA-4/combination therapy	34/5/5/21	544/92/155/324	0.529
RCC/NSCLC/melanoma/other	16/15/21/13	209/298/354/254	0.656
Diabetes/Thyroid/Other	3/28/0	40/138/11	0.097
Non-serious/Hospitalized/Life-threatened or disabled/Death	16/33/10/6	227/598/155/135	0.783

Categorical variants were compared using chi-square analysis, and continuous variants were compared using student’s *t* test. ICI regimens included anti-PD-1, anti-PD-L1, anti-CTLA4 monotherapy and combination therapy. Cancer types of the patients included RCC, NSCLC, malignant melanoma and other types of cancer. Accompanied endocrinopathies indicated ICI-related endocrinopathies besides PAI, including diabetes, thyroid dysfunctions and other kinds of endocrinopathies. Clinical outcomes included non-serious, hospitalized, life-threatened or disabled, and death. RCC, renal cell carcinoma; NSCLC, non-small-cell lung carcinoma

Among patients on PD-1 inhibitors, the incidence of PAI was 0.77% (578/74605), 0.60% (97/16071) for PD-L1 inhibitors, 0.68% (160/23445) for CTLA-4 inhibitors and 1.47% (345/23445) for combination therapy of anti-PD-1 + anti-CTLA-4. Patients on PD-1 inhibitors had a higher risk of PAI than those in the anti-PD-L1 group (χ^2^ = 5.14, *p* = 0.022) and showed a significantly lower risk of PAI than those in the combination therapy group (χ^2^ = 92.88, *p* < 0.001). There was no reported adrenal crisis in the FAERS.

### Demographic characteristics of ICI-PAI

Overall, 738 individuals (62.5%) were male, 359 (30.4%) were female and 83 subjects (7.1%) were not specified. Male individuals showed a higher risk of ICI-PAI [male vs. female, 1.17% (738/62594) vs. 0.94% (359/38179), χ^2^ = 12.55, *p* < 0.001], and a significant predisposition of men was still observed in those receiving anti-PD-1 therapy and the combination therapy of anti-PD-1 and anti-CTLA-4 [anti-PD-1 therapy, male vs. female, 0.93% (384/41282) vs. 0.58% (144/24673), χ^2^ = 23.36, *p* < 0.001; anti-PD-1 + anti-CTLA-4 therapy, 0.63% (54/8522) vs. 0.57% (35/6099), χ^2^ = 0.21, *p* = 0.647; anti-CTLA-4 therapy, 0.74% (95/12790) vs. 0.76% (56/7407), χ^2^ = 0.01, *p* = 0.15; anti-PD-1 + anti-CTLA-4 therapy, 1.60% (205/12790) vs. 1.67% (124/7425).

Regarding the age distribution, 439 (37.2%) patients were less than 65 years old, 588 (49.8%) were equal to or above 65 years old, and 153 (13.0%) were not specified. The incidence of ICI-PAI was significantly reduced in younger patients [age < 65 years, 439/36672 (1.20%)] compared with those older than 65 years [588/41679 (1.41%), *χ*^2^ = 6.89, *p* = 0.009]. Similar results between different age groups were observed in patients receiving anti-PD-1 therapy [< 65 vs. ≥ 65, 0.76% (171/22423) vs. 1.20% (330/27546), χ^2^ = 23.61, *p* < 0.001] and the combined therapy of anti-PD-1 and anti-CTLA-4 [< 65 vs. ≥ 65, 1.68% (142/8456) vs. 2.35% (169/7194), χ^2^ = 0.8.96, *p* = 0.003].

Among all patients with ICI-PAI on PD-1 inhibitors, 51.9% (300 of 578) were on nivolumab, 46.7% (270 of 578) were on pembrolizumab and 1.4% (8 of 578) were on cemiplimab. Of all 332 ICI-PAI patients on the combination therapy of anti-PD-1 + anti-CTLA-4, 94.3% (324 of 332) were on nivolumab + ipilimumab treatment, and 5.7% (8 of 332) were using the pembrolizumab + ipilimumab regimen.

Of all 1180 ICI-PAI patients, 225 (19.1%) patients were diagnosed with renal cell carcinoma, 313 (26.5%) with non-small cell lung carcinoma (NSCLC), 375 (31.8%) with malignant melanoma and 267 (22.6%) with other types of cancer, including squamous cell carcinoma of the head and neck (Table 2).

**Table 2:** Clinical characteristics of patients with ICI-related primary adrenal insufficiency

Characteristics	All (*n* = 1180)	Anti-PD-1 therapy (*n* = 578)	Anti-PD-L1 therapy (*n* = 97)	Anti-CTLA-4 therapy (*n* = 160)	Anti-PD-1 and anti-CTLA-4 therapy (*n* = 345)
Sex categories (*n*, %)					
Male	738 (62.5)	384 (66.4)	54 (55.7)	95 (59.4)	205 (59.4)
Female	359 (30.4)	144 (24.9)	35 (36.1)	56 (35.0)	124 (35.9)
Not specified	83 (7.1)	50 (8.7)	8 (8.2)	9 (5.6)	16 (4.6)
Age (years) (*n*, %)					
18–64	439 (37.2)	171 (29.6)	41 (43.3)	85 (53.1)	142 (41.2)
≥ 65	588 (49.8)	330 (57.1)	39 (40.2)	50 (31.3)	169 (49.0)
Not specified	153 (13.0)	77 (13.3)	17 (17.5)	25 (15.6)	34 (9.9)
Reporting year (*n*, %)					
2015 and before	134 (11.4)	18 (3.1)	0 (0)	93 (58.1)	26 (7.5)
2016	71 (6.0)	36 (6.2)	0 (0)	16 (10.0)	19 (5.5)
2017	139 (11.8)	78 (13.5)	15 (15.5)	17 (10.6)	29 (8.4)
2018	186 (15.8)	97 (16.8)	22 (22.7)	21 (13.1)	46 (13.3)
2019	276 (23.4)	160 (27.7)	20 (20.6)	10 (6.3)	86 (24.9)
2020	378 (32.0)	188 (32.5)	40 (41.2)	3 (1.9)	147 (42.6)
Cancer type (*n*, %)					
Renal cell carcinoma	225 (19.1)	90 (15.6)	17 (17.5)	4 (2.5)	114 (33.0)
Non-small-cell lung carcinoma	313 (26.5)	233 (40.3)	45 (46.4)	4 (2.5)	31 (9.0)
Malignant melanoma	375 (31.8)	90 (15.6)	4 (4.1)	129 (80.6)	152 (44.1)
Other types	267 (22.6)	165 (28.5)	31 (32.0)	23 (14.4)	48 (13.9)
Accompanied with other endocrinologic irAEs (*n*, %)					
Total	286 (24.2)	132 (22.8)	19 (19.6)	38 (23.8)	97 (28.1)
T1DM	43 (3.6)	21 (3.6)	0 (0)	1 (0.6)	21 (6.1)
Thyroid dysfunction	166 (14.1)	89 (15.4)	12 (12.4)	23 (14.4)	42 (12.2)
Other endocrinopathies *	77 (6.5)	22 (3.8)	7 (7.2)	14 (8.8)	34 (9.9)
Prognosis (*n*, %)					
Non-serious	243 (20.6)	122 (21.1)	27 (27.8)	45 (28.1)	49 (14.2)
Hospitalized	631 (53.5)	290 (50.2)	51 (52.6)	88 (55.0)	202 (58.6)
Life-threatened or disabled	165 (14.0)	92 (15.9)	6 (6.2)	10 (6.3)	57 (16.5)
Death	141 (11.9)	74 (12.8)	13 (13.4)	17 (10.6)	37 (10.7)

*Accompanied endocrinopathies besides PAI, T1DM or thyroid dysfunction

### The co-occurrence of other endocrinopathies in patients with ICI-PAI

The co-occurrence rate of endocrinopathies other than PAI was 24.2% (286/1180) in all patients receiving ICI therapy. The most common type of accompanying endocrinopathy was thyroid dysfunction (166, 14.1%), followed by ICI-related type 1 diabetes (43/1180, 3.6%). Among the different regimens, the highest occurrence rate of accompanying endocrinopathy was 28.1% (97/345) in patients on PD-1 inhibitor + CTLA-4 inhibitor treatment, followed by 23.8% (38/160) in patients on CTLA-4 inhibitors and 22.8% (132/578) in those on PD-1 inhibitors, and the lowest rate was 19% (19/97) in those on PD-L1 inhibitors. There was no significant difference in other ICI-associated endocrinopathies between patients on anti-CTLA-4 therapy and those on the combination therapy of anti-PD-1 + anti-CTLA-4 (χ^2^ = 1.064, *p* = 0.302). An increasing trend was discovered among patients on the combination therapy compared with those on PD-1 inhibitors (χ^2^ = 3.227, *p* = 0.072). Additionally, no significant difference was found in the occurrence rate of other ICI-associated endocrinopathies between patients on anti-PD-L1 therapy and those on anti-PD-1 therapy (χ^2^ = 0.043, *p* = 0.836).

We further analysed the subtypes of accompanying ICI-related endocrine diseases. In ICI-PAI patients with T1DM, only one patient with PAI on anti-CTLA-4 monotherapy developed T1DM (1/160). No significant difference was discovered in the incidence of T1DM in PAI patients on PD-1 inhibitors and the combination therapy of anti-PD-1 + anti-CTLA-4 (χ^2^ = 2.995, *p* = 0.084). Additionally, no difference in the distribution of accompanying thyroid dysfunction was found among the four regimens (*χ*^2^ = 1.860, *p* = 0.602).

### Distribution of ICI-PAI based on the reporting year

Overall, the proportion of ICI-PAI to all reported ICI-related adverse events significantly increased from 0.67% (71/10,590) in 2016 to 1.25% (378/30,246) in 2020 (*χ*^2^ = 24.21, *p* < 0.001, Bonferroni corrected, Table 2). In the anti-PD1 therapy subgroup, the incidence of PAI significantly increased from 0.48% (18/3788) in 2015 to 0.98% (188/19254) in 2020 (χ^2^ = 8.98, *p* = 0.003, Bonferroni corrected). The incidence of PAI in subjects on anti-PD-L1 therapy was 0.90% (15/1662) in 2017 and it declined to 0.64% (40/6221) in 2020 (*χ*^2^ = 1.27, *p* = 0.259, Bonferroni corrected). The reported number of ICI-PAI among patients on anti-CTLA-4 monotherapy declined over time, from 1.50% (93/6200) in 2015 and before to 0.06% (3/4771) in 2020 (Fisher’s exact test, *p* < 0.001, Bonferroni corrected). A significant increase was discovered in the incidence of PAI in individuals receiving the combined regimen of anti-PD-1 and anti-CTLA-4 therapy, from 0.42% (26/6200) in 2015 and before to 3.08% (147/4771) in 2020 (*χ*^2^ = 123.08, *p* < 0.001, Bonferroni corrected).

### Clinical outcomes in patients with ICI-PAI

In terms of prognosis, 937 (79.4%) cases were severe, and 243 (20.6%) were nonserious. Among the severe cases, 631 (53.5%) were hospitalized, 165 (14.0%) were life-threatening or disabled, and 141 (11.9%) were deaths due to various causes. The risk of severe adverse events (AEs) increased in patients on the combination therapy of anti-PD-1 and anti-CTLA-4 compared with those on PD-1 inhibitors [OR 1.616, 95% CI (1.125–2.322), *p* = 0.009]. A tendency towards a lowered risk of severe AEs was observed in patients on CTLA-4 inhibitors compared with those on PD-1 inhibitors (*χ*^2^ = 3.53, *p* = 0.060), while the occurrence of severe AEs was insignificant in patients on the other monotherapy regimens (anti-PD-1 vs. anti-PD-L1, *χ*^2^ = 2.19, *p* = 0.139). Patients with PAI on anti-PD-1 therapy showed a similar risk of death from all possible causes, including advanced malignancy, compared with other regimens (anti-PD-L1, *χ*^2^ = 0.03, *p* = 0.871; anti-CTLA-4, *χ*^2^ = 0.55, *p* = 0.458; anti-PD-1 + anti-CTLA-4, *χ*^2^ = 0.88, *p* = 0.348).

Further analysis was conducted to explore risk factors correlated with the prognosis, including age, sex, cancer type, other endocrinopathies and body weight, in patients on different regimens. Body weight was negatively associated with the risk of death in the study population [*p* = 0.033 for the regression model; B = – 0.017, OR 0.984, 95% CI (0.969–0.998), *p* = 0.029], while different regimens, sex, age and endocrine comorbidities showed insignificant influences on the regression model. Similarly, increased body weight reduced the risk of death in patients on the combination therapy of anti-PD-1 and anti-CTLA-4 [*p* = 0.031 for the regression model; B = – 0.041, OR 0.960, 95% CI (0.932–0.989), *p* = 0.007]. In patients on CTLA-4 inhibitors, the cancer type of melanoma was positively associated with the risk of severe cases [B = 3.607, OR 36.873, 95% CI (1.833–741.761), *p* = 0.018]. In the subgroup of PD-1 inhibitors, the comorbidity of ICI-related thyroid dysfunction (ICI-TD) was positively related to the risk of a severe clinical prognosis [*p* = 0.049 for the regression model; B = 1.225, OR 3.508, 95% CI (1.074–11.458), *p* = 0.038].

## Discussion

To our knowledge, this study represents the most recent and largest report of the clinical descriptions and characteristics of ICI-associated PAI using individual adverse event reports from the FDA FAERS database. Although PAI is a rare adverse event, it calls for emergent reorganization and treatment due to its possibility of adrenal crisis and death. Our study revealed an increasing incidence of ICI-associated PAI from 2016 to 2020. The increasing trend of incidence of ICI-PAI over time may be explained by a more prevalent application of ICI therapy and a more profound understanding of its AEs by clinicians.

In our study, the incidence of ICI-PAI was 1.03% (1180 to 114,121) in all reported cases of ICI-related adverse events and between 0.60 and 0.77% in individuals on monotherapy, which was in accordance with an incidence of less than 1% in a previous study [2]. Our research indicated an incidence of ICI-PAI of 1.47% in patients on the combination therapy of ipilimumab and nivolumab. This was lower than the incidence reported for patients on combination therapy, which was 4–8% [8]. In randomized clinical trials and meta-analyses, the incidence of ICI-PAI was up to 2.43% [9]. Since a proportion of nonserious cases may not be reported in real-world practice, this may result in an underestimated incidence of ICI-PAI in the FAERS database.

Our study suggested that ICI-PAI had a distinct sex and age distribution compared with other irAE endocrinopathies or autoimmune PAI. Of all 1180 cases of ICI-PAI, 62.5% were men in our study population. A similar result was found in a study using data from VigiBase, the World Health Organization pharmacovigilance database, in which 54.4% of all 451 cases of PAI-irAE occurred in men [10]. However, some studies revealed a female predominance of ICI-induced endocrinopathies [11], and different endocrinologic irAEs presented with diverse gender predominances. A population study using data from the FAERS consistently indicated that women had significantly higher reporting frequencies of both hypothyroidism and hyperthyroidism, while hypophysitis showed a male-dominated disparity [12].

Our study found a significantly higher susceptibility to ICI-PAI in patients older than 65 years, and this predominance of elderly patients was seen in individuals receiving anti-PD-1 therapy or the combined therapy of anti-PD-1 and anti-CTLA-4. There were no reports of patients under the age of 18 who developed ICI-induced PAI in the FAERS since the prevalence of lung cancer, renal cell carcinoma and melanoma is very low in the paediatric population.

Regarding the co-occurrence of endocrinopathies, 24.2% of cases with ICI-PAI developed polyendocrinopathies. Ipilimumab monotherapy or the combined therapy of anti-CTLA-4 and anti-PD-1 resulted in the highest risk of polyendocrinopathies, including PAI, followed by anti-PD-1 monotherapy, which was consistent with other researchers [13]. This could be explained by the possible destructive potential of ipilimumab and its combination therapy on endocrine glands, including thyroid glands and pancreas *β* cells. More frequent surveillance of thyroid and adrenal function should be implemented in patients on CTLA-4 inhibitors or their combination therapy.

In our study, 79.4% of ICI-PAI cases were severe, and the mortality rate was 11.9% due to various causes. A previous study reported a severe adverse event rate of 91.1% and a mortality rate of 7.3% in PAI-irAE patients using VigiBase, in which the severe adverse event had a broadened definition of the outcome of death, being life-threatening, hospitalization, disability or any other medically important conditions [10]. In both studies, ICI-PAI could lead to a high proportion of serious outcomes, which could be explained by damage to the adrenal gland and acute insufficiency of serum cortisol. Our study suggested that the risk of serious events was increased in the subgroup of the combination therapy of anti-PD-1 and anti-CTLA-4, and the risk of death or severe clinical outcome was insignificant for the different monotherapies. Some researchers concluded that patients on PD-1 inhibitors had increased mortality after ICI monotherapy [14], and combination therapy with ICIs was proven to be a positive factor for poor clinical outcomes [15, 16].

In our study, further analysis of prognostic factors suggested that lower body weight could lead to a higher risk of death in the study population. Cancer-associated weight loss indicates a poor prognosis; thus, a higher BMI in patients with malignancy and slower weight loss during treatment suggests a better survival rate [17]. The link between body weight and prognosis in patients receiving ICI therapy was first proven in our study. In the subgroup of PD-1 inhibitors, the comorbidity of ICI-related thyroid dysfunction in ICI-PAI patients revealed a positive correlation with severe clinical outcomes, while no association was discovered between ICI-TD and the risk of death. A prospective study and an observational study indicated that the overall survival was significantly longer in patients on ICI treatment after developing ICI-related thyroid dysfunction, and the prognosis was even better in patients with overt hypothyroidism [18–20]. One possible explanation was that patients who developed irAEs might also have a preferable treatment response, while irAEs alone could bring about poor clinical outcomes. Therefore, more attention should be given to the monitoring of irAEs, especially in patients with a lower body mass during ICI treatment.

No cases of the adrenal crisis were reported by the FAERS. Little is known about ICI-induced adrenal crisis, a life-threatening disease. Regular measurement of serum cortisol and adrenocorticotropin could be recommended for patients on ICI treatment who present with fatigue or nausea to facilitate an early diagnosis [21].

As a retrospective study, referral bias could result in limitations of this study. Since a proportion of nonserious cases may be neglected and not reported in the FAERS database, it could lead to bias in the analysis of the incidence and prognosis of the disease. More importantly, the exact levels of hormones, including adrenocorticotropic hormone (ACTH) and renin, were not available in the FAERS, which is critical to the differential diagnosis of primary or secondary adrenal insufficiency, and it inevitably generates bias in the estimate of the incidence of ICI-PAI. Although the reaction terms, including secondary or pituitary adrenal insufficiency and hypophysitis, were excluded from our study population, the data of FAERS reporting are voluntary from endocrinologists as well as oncologists and general practitioners, and it is unrealistic to confirm the diagnosis of PAI or central adrenal insufficiency by all reporters. Therefore, we categorized the ICI-PAI cases from the FAERS into “confirmed PAI” and “suspected PAI” based on the reported terms of hyperkalaemia and increased ACTH or renin. There was no significant difference in the demographic data, ICI regimens or clinical outcomes between patients with “confirmed PAI” and those with “suspected PAI”. Based on these results, we conducted further statistical analysis in all reported 1180 cases of ICI-PAI. Unfortunately, a large proportion of the reported cases failed to provide the level of ACTH and renin or whether there was hyperkalaemia, which might result in an overestimated incidence of ICI-PAI. In addition, our study focused on ICI-mediated newly diagnosed PAI instead of other causes of PAI, including bilateral adrenal metastasis or haemorrhage, which introduced bias in the data analysis.

## Conclusion

ICI-associated PAI is a rare but important irAE. Male and elderly patients have a higher risk of ICI-PAI. The combined regimen of ipilimumab and nivolumab results in a higher possibility of polyendocrinopathies, including PAI. Awareness among health care professionals is critical when patients with a lower body weight develop PAI, which indicates a higher risk of a poor clinical outcome.

## Data Availability

The datasets used and/or analysed during the current study are available from the corresponding author on reasonable request.

## Abbreviations

ICI: Immune checkpoint inhibitor
PD-1: Programmed cell death protein-1
PD-L1: Programmed death-ligand 1
CTLA-4: Cytotoxic T-cell-associated protein-4
irAEs: Immune-related adverse events
AE: Adverse event
PAI: Primary adrenal insufficiency
FAERS: FDA Adverse Event Reporting System
T1DM: Type 1 diabetes mellitus

## References

[1] Brahmer JR, Lacchetti C, Schneider BJ (2018). National comprehensive cancer network management of immune-related adverse events in patients treated with immune checkpoint inhibitor therapy: American society of clinical oncology clinical practice guideline. J Clin Oncol.

[2] Barroso-Sousa R, Barry WT, Garrido-Castro AC (2018). Incidence of endocrine dysfunction following the use of different immune checkpoint inhibitor regimens: a systematic review and meta-analysis. JAMA Oncol.

[3] Bancos I, Hahner S, Tomlinson J (2015). Diagnosis and management of adrenal insufficiency. Lancet Diabetes Endocrinol.

[4] Byun DJ, Wolchok JD, Rosenberg LM (2017). Cancer immunotherapy- immune checkpoint blockade and associated endocrinopathies. Nat Rev Endocrinol.

[5] Yang JC, Hughes M, Kammula Y (2007). Ipilimumab (anti-CTLA4 antibody) causes regression of metastatic renal cell cancer associated with enteritis and hypophysitis. J Immunother.

[6] Traner H, Hulse P, Higham CE (2016). Hyponatremia secondary to nivolumab-induced primary adrenal failure. Endocrinol Diabetes Metab Case Rep..

[7] Min L, Ibrahim N (2013). Ipilimumab-induced autoimmune adrenalitis. Lancet Diabetes Endocrinol.

[8] Cukier P, Santini FC, Scaranti M (2017). Endocrine side effects of cancer immunotherapy. Endocr Relat Cancer.

[9] Lu J, Li L, Lan Y (2019). Immune checkpoint inhibitor-associated pituitary-adrenal dysfunction: a systematic review and meta-analysis. Cancer Med.

[10] Grouthier V, Lebrun-Vignes B, Moey M (2020). Immune checkpoint inhibitor-associated primary adrenal insufficiency: WHO VigiBase report analysis. The Oncologists.

[11] Triggianese P, Novelli L, Galdiero MR (2020). Immune checkpoint inhibitors-induced autoimmunity: the impact of gender. Autoimmun Rev.

[12] Zhai Y, Ye X, Hu F (2019). Endocrine toxicity of immune checkpoint inhibitors: a real-world study leveraging US food and drug administration adverse events reporting system. J Immuno Therapy Cancer.

[13] Xu C, Chen YP, Du XJ (2018). Comparative safety of immune checkpoint inhibitors in cancer: systematic review and network meta-analysis. BMJ.

[14] Sun X, Roudi R, Dai T (2019). Immune-related adverse events associated with programmed cell death protein-1 and programmed cell death ligand 1 inhibitors for non-small cell lung cancer: a PRISMA systematic review and meta-analysis. BMC Cancer.

[15] Wolchok JD, Chiarion-Sileni C, Gonzalez R (2017). Overall survival with combined nivolumab and ipilimumab in advanced melanoma. N Engl J Med.

[16] Arnaud-Coffin P, Maillet D, Gan HK (2019). A systematic review of adverse events in randomized trials assessing immune checkpoint inhibitors. Int J Cancer.

[17] Martin L, Senesse P, Gioulbasanis I (2015). Diagnostic criteria for the classification of cancer- associated weight loss. J Clin Oncol.

[18] Osorio JC, Ni A, Chaft JE (2017). Antibody-mediated thyroid dysfunction during T-cell checkpoint blockade in patients with non-small-cell lung cancer. Ann Oncol.

[19] Kim HI, Kim M, Lee SH (2017). Development of thyroid dysfunction is associated with clinical response to PD-1 blockade treatment in patients with advanced non-small cell lung cancer. Onco Immunol.

[20] Yamauchi I, Yasoda A, Matsumoto S (2019). Incidence, features, and prognosis of immune-related adverse events involving the thyroid gland induced by nivolumab. PLoS ONE.

[21] Reznik Y, Barat P, Bertherat J (2018). SFE/SFEDP adrenal insufficiency French consensus: introduction and handbook. Ann Endocrinol (Paris).

